# Beef Heart Inclusion Impacts Physiochemical Stability of Vacuum-Packaged Ground Beef During Refrigerated Storage

**DOI:** 10.3390/foods15203722

**Published:** 2026-10-19

**Authors:** Savannah L. Douglas, Autumn L. Armaly, Rilee A. Bennett, Xenia M. Murillo-Contreras, Don R. Mulvaney, Soren P. Rodning, Jase J. Ball, Jason T. Sawyer

**Affiliations:** 1 Department of Animal Sciences, Auburn University, Auburn, AL 36849, USA; sld0060@auburn.edu (S.L.D.); ala0084@auburn.edu (A.L.A.); rzb0145@auburn.edu (R.A.B.); mulvadr@auburn.edu (D.R.M.); rodnisp@auburn.edu (S.P.R.); jjb0127@auburn.edu (J.J.B.); 2 Department of Poultry Sciences, Auburn University, Auburn, AL 36849, USA

**Keywords:** color, beef, beef heart, ground beef, TBARS, refrigerated storage, retail storage, shelf-life, vacuum packaging

## Abstract

Edible beef heart may improve whole-animal utilization while enhancing the nutritional value of ground beef; however, its effects on refrigerated storage stability remain unclear. This study evaluated the influence of beef heart on the shelf-life characteristics of vacuum-packaged ground beef. Ground beef was produced in three batches per treatment from beef trimmings (80% lean and 20% fat). Treatments consisted of control (0% heart), 6% heart, or 12% heart. Samples were vacuum packaged and evaluated on days 0, 5, 10, and 15 for instrumental surface color, myoglobin forms, thiobarbituric acid reactive substances (TBARS), and pH. Treatment × storage day interactions were observed for pH (*p* < 0.05), TBARS (*p* < 0.05), instrumental color (*p* < 0.05), and calculated myoglobin forms (*p* < 0.05). Including beef heart in ground beef altered surface color characteristics, resulting in lower a* values (*p* < 0.05) and greater hue angle (*p* < 0.05) on day 0. TBARS values increased with heart addition throughout storage, with the 12% heart treatment exhibiting the greatest TBARS values (*p* < 0.05). Beef heart inclusion altered some physicochemical characteristics, but these changes did not compromise product stability during 15 days of refrigerated vacuum storage.

## 1. Introduction

Development of value-added meat products containing edible beef variety meats provides an opportunity to improve carcass utilization while increasing the nutritional and economic value of foods already familiar to consumers [1]. Although skeletal muscle represents the primary portion of meat marketed for human consumption in the United States, edible organs such as the heart, liver, and kidney contain high-quality protein, vitamins, minerals, and other bioactive compounds that support their use as a nutrient-dense food source [1,2,3]. Since market value and human consumption of these tissues vary among countries and cultures, adding beef organs into commonly consumed products, such as ground beef, offers a practical strategy to increase their utilization without requiring consumers to prepare or consume organ meats as separate products [1].

Beef heart is most suitable for formulating into ground beef as it is composed of striated muscle and can be processed using manufacturing procedures typically used on skeletal muscle fabrication [2]. Beef heart provides substantial concentrations of protein and is an excellent source of iron, zinc, selenium and phosphorus, although its composition and physicochemical characteristics differ from those of conventional skeletal muscle [2]. These compositional differences may influence the color, moisture retention, pH, and oxidative stability of products containing beef heart [2]. Determining whether beef heart can be incorporated without compromising product quality requires evaluation beyond initial composition, processing characteristics, or consumer acceptance [2]. Stability of the ground product during refrigerated storage must be evaluated before organ-enhanced ground beef can be considered a commercially viable consumer product available in a retail setting [4].

Shelf life is a critical determinant of the economic feasibility of fresh meat products [5]. During refrigerated storage and retail display, fresh ground beef undergoes progressive changes in surface color, TBARS, moisture retention, microbial populations, and other physicochemical characteristics [5,6]. These changes may reduce consumer acceptability and result in retail markdowns or product disposal [6]. Ground beef is susceptible to deterioration as grinding disrupts the structure of the muscle, incorporates oxygen, redistributes fat, and increases the surface area available for oxidative reactions [5,6]. For consumers, surface color is influential since consumers often associate a bright, cherry-red appearance with freshness and wholesomeness [6]. As storage progresses, oxidation of myoglobin to metmyoglobin produces brown discoloration that may decrease purchase intent even when the product remains suitable for consumption [5,6].

Meat color stability is closely associated with the oxidation state of myoglobin and the oxidative environment surrounding the pigment [5,6]. In oxygenated packaging, myoglobin initially forms oxymyoglobin, producing the bright-red appearance preferred by many consumers [6]. Continued exposure to oxygen and light can promote metmyoglobin formation and surface discoloration [5,6]. The literature reports that redness and oxymyoglobin decrease with metmyoglobin formation and an increase in thiobarbituric acid reactive substances during the refrigerated display of ground beef packaged in oxygen-permeable polyvinyl chloride film [7]. Thus, surface color and lipid oxidation are primary indicators of oxidative deterioration during fresh meat storage [5,7].

In addition, pH of meat influences protein functionality, water-holding capacity, microbial growth, and state of myoglobin [5,6]. Differences in pH can alter the storage behavior of ground beef formulated with heart [2]. Evaluation of pH alongside surface color and TBARS can provide a more comprehensive evaluation of the physicochemical stability of ground beef containing beef heart [4].

Previous studies have demonstrated that vacuum packaging improves color stability and reduces oxidative deterioration in conventional ground beef and beef steaks [8]. However, these findings cannot be projected on formulations containing edible beef heart as cardiac muscle differs from skeletal muscle in its composition and metabolic characteristics. Beef heart contains greater concentrations of myoglobin and heme iron than skeletal muscle [2]. These characteristics may alter myoglobin chemistry, TBARS, and physicochemical stability during storage. While beef heart can improve the nutritional profile of ground beef without substantially reducing consumer acceptability, it is unknown whether these formulations exhibit storage characteristics comparable to conventional ground beef [2]. This represents an important barrier prior to commercial adoption. Fresh ground beef products must maintain acceptable color and oxidative stability throughout refrigerated distribution and retail storage to minimize product loss and maintain consumer acceptance. If incorporation of beef heart accelerates color change or TBARS, its practical application may be limited despite the nutritional and sustainability advantages. If organ-enhanced ground beef has an extended or comparable shelf life to conventional ground beef, then beef heart may serve as a viable ingredient for developing value-added ground beef products that improve whole-animal utilization while maintaining product quality and retail stability. Our previous studies have demonstrated that ground beef formulated with 6% and 12% beef heart exhibits acceptable consumer acceptance [9,10]. However, the refrigerated storage stability of these formulations remains unknown. Therefore, the objective of this study was to determine the effects of beef heart inclusion on physicochemical shelf-life characteristics of vacuum-packaged ground beef during 15 days of refrigerated storage.

## 2. Materials and Methods

### 2.1. Raw Materials, Processing and Packaging

Beef trimmings with 80% lean and 20% fat according to supplier specifications (69.85 kg; USDA Institutional Meat Purchasing Specifications #138) and beef heart (12.57 kg; USDA Institutional Meat Purchasing Specifications #1723) were sourced from a commercial meat supplier (Capital Farms, Wickenburg, AZ, USA) and transported under refrigerated conditions to the Lambert-Powell Meats Laboratory (Auburn, AL, USA). Raw materials were commercially sourced. Therefore, individual animal identity and carcass origin were not available for matching beef heart and trimmings.

Three 7.75 kg batches for each treatment were manufactured. Treatments consisted of control (0% beef heart), 6% beef heart, and 12% beef heart. Beef heart inclusion was calculated as a percentage of the target formulation weight and incorporated on a weight-for-weight replacement basis for beef trimmings. Accordingly, 6% and 12% of the beef trimmings were replaced with an equivalent weight of beef heart for the respective treatments, while the control consisted entirely of beef trimmings. This maintained an equivalent target formulation weight among treatments. Inclusion rates of 6% and 12% beef heart inclusion levels were selected based on previous research in which incorporation at 15% negatively affected product quality and consumer acceptability, supporting evaluation of lower inclusion levels [9]. These inclusion levels were also consistent with formulations previously evaluated [10]. Beef trimmings were coarse ground through a 9.525 mm plate (SPECO 400, Schiller Park, IL, USA) using a commercial grinder (Model AFMG-48, Biro Manufacturing Company, Marblehead, OH, USA). Beef heart was separately processed through the same 9.525 mm grinding plate prior to incorporation into treatment formulations. Following organ addition, treatments were mixed for 3 min and subsequently ground through a 3.18 mm plate (SPECO 400, Schiller Park, IL, USA) fitted with a bone eliminator attachment (SPECO 400, Schiller Park, IL, USA). Ground beef mixtures were portioned into 453.59 g bricks (n = 17 bricks/batch/treatment) using a vacuum filler (Vemag HP1, Reiser, Canton, MA, USA). Five packages were designated for repeated nondestructive instrumental color measurements throughout storage, while three independent packages were designated for destructive sampling at each storage day (days 0, 5, 10, and 15). Following portioning, samples were packaged using a thermoforming packaging system (Optimus OL0924, Variovac, Zarrentin, Germany) with a forming film oxygen transmission rate of 0.4 cc/m^2^/24 h and a non-forming film oxygen transmission rate of 1.0 cc/m^2^/24 h (WINPAK Ltd., Winnipeg, MB, Canada).

### 2.2. pH Analysis

On each sampling day, ground beef pH was determined using a 2 g sample combined with 20 mL of deionized water in a plastic centrifuge tube. The mixture was homogenized for 45 s using a Kinematica homogenizer (CH-6010, Brinkmann Instruments, Inc., Westbury, NY, USA). Following homogenization, pH was recorded using a glass-electrode pH meter (Model HI99163, Hanna Instruments, Woonsocket, RI, USA). Prior to sample analysis, the instrument was calibrated at pH 4.0 and 7.0 using two-point standard buffers (Thermo Fisher Scientific, Chelmsford, MA, USA).

### 2.3. Instrumental Color

Instrumental surface color was evaluated on ground beef samples (n = 5/batch/treatment) using a HunterLab EZ colorimeter (Model 45/0 LAV, Hunter Associates Laboratory Inc., Reston, WV, USA) in accordance with American Meat Science Association guidelines [11]. Before sample evaluation, the instrument was standardized using black and white calibration tiles according to the manufacturer’s instructions. Measurements were collected using illuminant A, a 10° standard observer, and a 31.88 mm aperture. Three readings were obtained from each sample and averaged for statistical analysis.

Instrumental color measurements were used to obtain L*, a*, and b* values and to calculate additional indices of color stability. Hue angle (°) was calculated as tan^−1^(b*/a*), while chroma (C*) was calculated as √(a*^2^ + b*^2^). Spectral reflectance was recorded from 400 to 700 nm, and the 630:580 nm reflectance ratio was used as an indicator of the change from red to brown surface color. Relative proportions of deoxymyoglobin (DMb), metmyoglobin (MMb), and oxymyoglobin (OMb) were calculated from spectral measurements according to equations provided in the AMSA Meat Color Measurement Guidelines [11]: DMb (%) = 2.375 × [1 − ((A473 − A730)/(A525 − A730))] × 100; MMb (%) = {1.395 − [(A572 − A730)/(A525 − A730)]} × 100; OMb (%) = 100 − (MMb% + DMb%).

### 2.4. Thiobarbituric Acid-Reactive Substances

Ground beef samples (n = 3/batch/treatment/day) were analyzed for thiobarbituric acid-reactive substances (TBARS) throughout the 15-day refrigerated storage period according to a previously described method [12]. For each sample, 2.0 ± 0.5 g of ground beef was analyzed in duplicate. Samples were combined with 8 mL of cold (1 °C) 50 mM phosphate buffer containing 0.1% ethylenediaminetetraacetic acid (EDTA), 0.1% n-propyl gallate, and 2 mL trichloroacetic acid (Sigma-Aldrich, Saint Louis, MO, USA), followed by homogenization using a Polytron homogenizer (Kinematica Polytron, CH-6010, Malters, Switzerland).

Following homogenization, the mixture was passed through Whatman No. 1 filter paper into borosilicate glass tubes. Two 2 mL aliquots of the resulting filtrate were transferred to separate 10 mL test tubes and combined with 2 mL of 0.02 M 2-thiobarbituric acid reagent (BeanTown Chemical, Hudson, NH, USA). Tubes were heated in a 100 °C water bath for 20 min and subsequently cooled in an ice bath for 15 min. Sample absorbance was then determined at 533 nm using a spectrophotometer (VWR UV-1600, VWR International, Radnor, PA, USA). Absorbance values were converted to TBARS concentrations using a factor of 12.21, and results were expressed as mg malonaldehyde/kg fresh meat. The conversion factor was previously established from a standard curve generated using known concentrations of malonaldehyde [12].

### 2.5. Statistical Analysis

This study was conducted as a randomized complete block design with production batch serving as the blocking factor. Three independent batches were produced within each treatment. Data analysis was performed using the GLIMMIX procedures of SAS (version 9.2; SAS Inst., Cary, NC, USA). Least-squares means were generated for treatment, storage day, and the treatment × storage day interaction, and results are presented as least-squares means ± SEM (standard error of the mean). Pairwise comparisons among least-squares means were conducted using the PDIFF option. When a significant treatment × storage day interaction was detected, pairwise comparisons of the interaction least-squares mean were used to evaluate treatment differences within individual storage days. Statistical significance was established at *p* ≤ 0.05.

## 3. Results

### 3.1. pH Values

**Figure 1:**
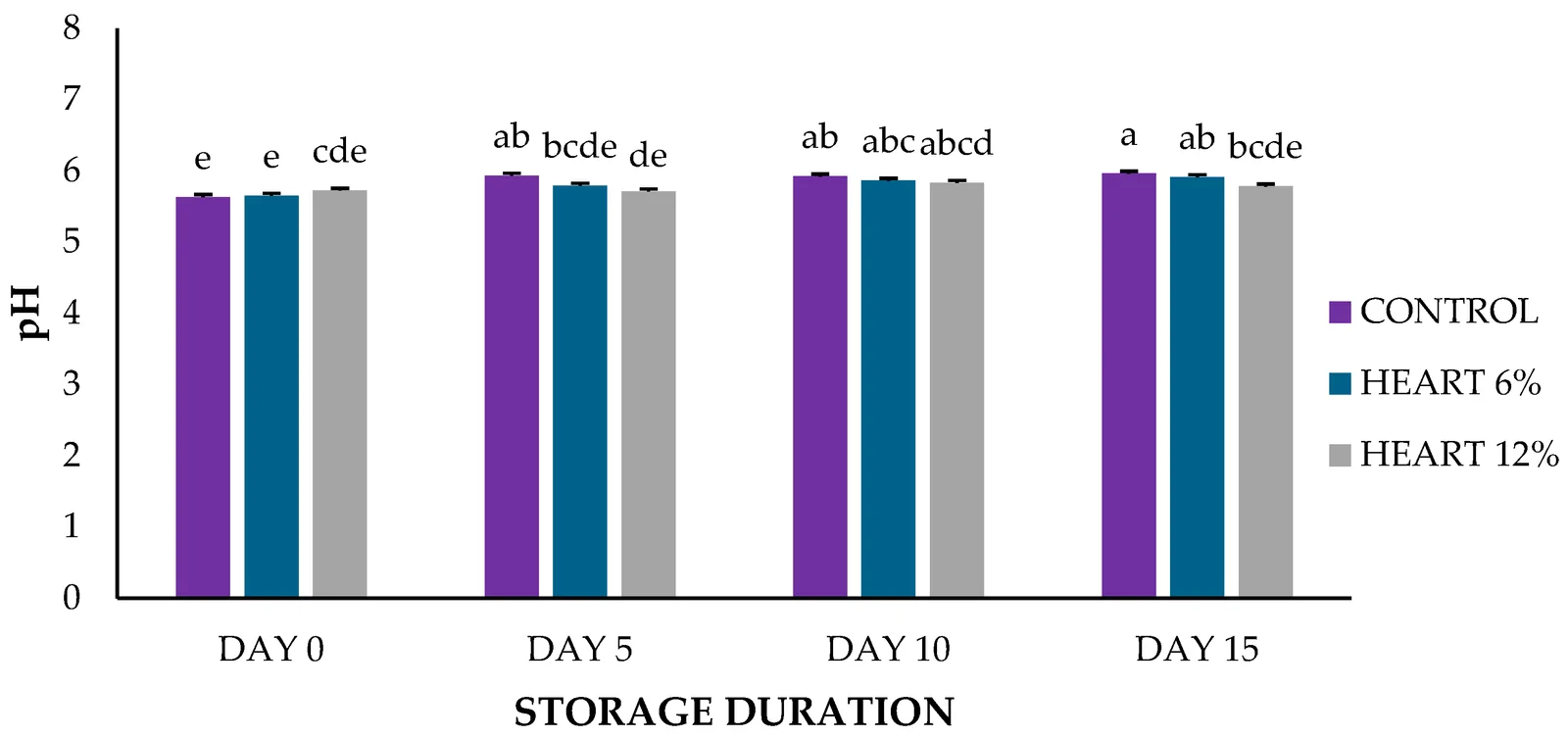
Interaction of ground beef formulation treatment × storage duration on pH. ^a–e^ Bars without common superscripts differ (*p* < 0.05).

Measurement of product pH can reflect biochemical and microbial changes that occur during refrigerated storage [13,14]. Changes in pH can influence meat color, water-holding capacity, and overall product stability [15]. pH results from the current study are presented in Figure 1. A treatment × storage day interaction influenced the pH of ground beef in the current study (*p* < 0.05). At the beginning of storage (day 0), pH did not differ among treatments (*p* > 0.05) indicating that beef heart inclusion had no immediate effect on product pH. As storage progressed, patterns emerged among formulations. Control exhibited the greatest increase in pH by day 5 and remained higher than the 12% beef heart treatment on both day 5 (*p* < 0.05) and day 15 (*p* < 0.05). Greater pH observed in control compared with the 12% heart treatment on days 5 and 15 may reflect differences in the biochemical characteristics of the formulations. Changes in pH during refrigerated storage have been associated with postmortem biochemical processes, including protein degradation and the accumulation of basic nitrogen-containing compounds [16,17]. It is plausible that incorporation of beef heart may have influenced these processes. In contrast, the 12% beef heart treatment remained relatively stable throughout storage, while the 6% beef heart formulation showed a more gradual increase in pH over time. Although the control tended to have a greater pH than the 6% treatment on day 5, this difference was not significant (*p* > 0.05), and no differences were detected between the 6% and 12% formulations on either day 5 (*p* > 0.05) or day 15 (*p* > 0.05).

Previous research demonstrates that the use of beef heart in ground beef increased moisture and reduced fat without affecting initial pH [10]. In the current study, no treatment differences in pH were observed at the beginning of storage indicating that beef heart incorporation did not alter the initial pH of the formulations. During refrigerated storage, increases in pH have been attributed to postmortem biochemical changes and the accumulation of basic nitrogen-containing compounds produced through protein degradation and microbial activity [16,17]. Changes in pH during storage have the potential to influence surface color [15,18]. In the current study, pH changed throughout storage and treatment differences were detected on days 5 and 15. However, these changes were not accompanied by consistent differences in instrumental surface color. A lower pH observed in treatments containing heart on days 5 and 15 may be associated with differences in glycogen availability and glycolytic activity. Glycogen serves as a primary substrate for postmortem glycolysis, and greater glycogen availability can promote glycolytic flux and lactate formation, contributing to a greater decline in pH [19]. Although glycogen concentration was not measured in the present study, differences in the metabolic characteristics of cardiac and skeletal muscle may have contributed to the observed treatment differences in pH [19]. These findings along with the current study results indicate that beef heart incorporation altered the pattern of pH change during refrigerated storage without producing changes in quality attributes.

### 3.2. Instrumental Surface Color

It is well known that consumers depend on visual color to determine the quality and wholesomeness of a product when selecting fresh proteins in the retail setting. Therefore, measurement of color is crucial in new product development or packaging studies. During storage, color was measured every 5 days for 15 days of storage. A treatment × storage day interaction was observed for L* (*p* < 0.05), a* (*p* < 0.05), b* (*p* < 0.05), hue angle (*p* < 0.05), and red-to-brown ratio (*p* < 0.05). Chroma was not affected by this interaction (*p* > 0.05); however, storage duration (day) significantly influenced the saturation of the samples (*p* < 0.05).

**Figure 2:**
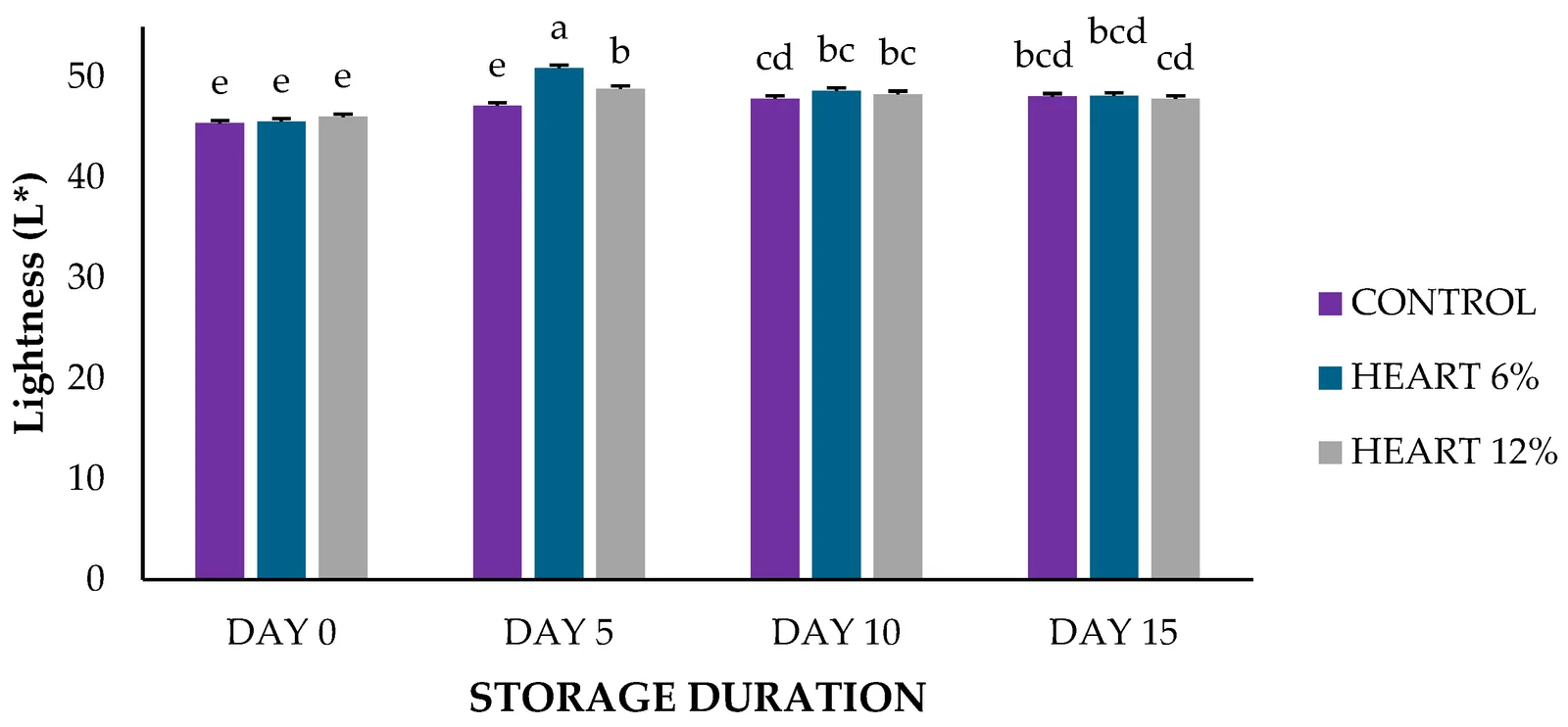
Interaction of ground beef formulation treatment × storage duration on lightness (L*). ^a–e^ Bars without common superscripts differ (*p* < 0.05).

L* values are presented in Figure 2. On day 0, L* values were similar between treatments (*p* > 0.05). On day 5, heart 6% exhibited greater L* values than both heart 12% and control (*p* < 0.05). No treatment differences were detected on day 10 (*p* > 0.05) or day 15 (*p* > 0.05). Lightness (L*) is influenced by factors such as light scattering, surface moisture, protein structure, and package-to-product contact and is not a direct measurement of myoglobin oxidation [20]. Treatment differences were transient as differences were observed on day 5 but were not detected on days 0, 10 or 15. Similar changes have been reported in vacuum-packaged ground beef during refrigerated storage [8,21].

**Figure 3:**
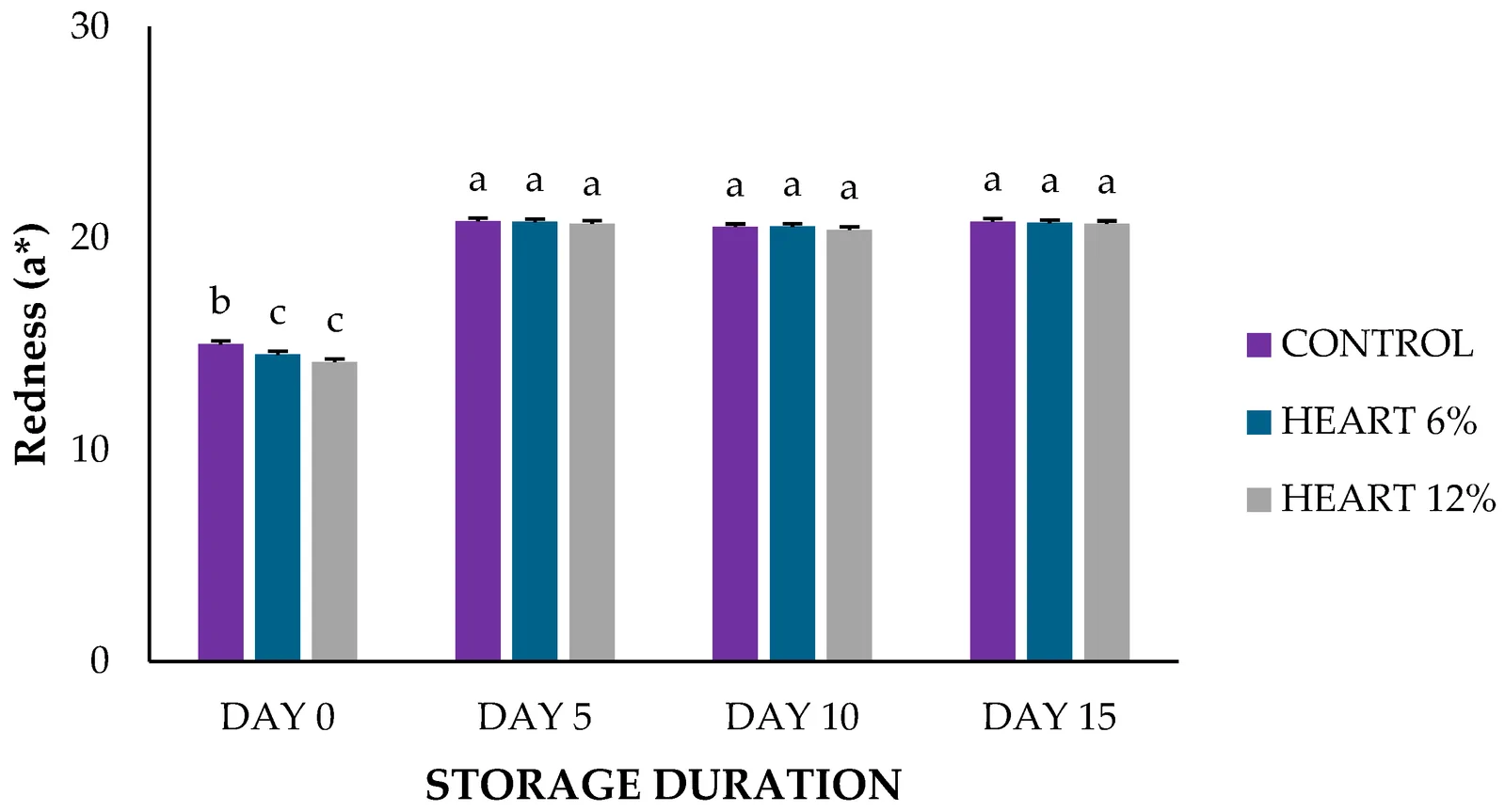
Interaction of ground beef formulation treatment × storage duration on redness (a*) values. ^a–c^ Bars without common superscripts differ (*p* < 0.05).

Surface redness (a*) was affected by a treatment × storage day interaction (*p* < 0.05; Figure 3). On day 0, control exhibited a greater a* value than both treatments containing heart (*p* < 0.05). Redness (a*) values increased in all treatments by day 5 with no treatment differences detected on day 5 (*p* > 0.05), day 10 (*p* > 0.05), or day 15 (*p* > 0.05). These findings suggest that beef heart altered the initial a* value of ground beef immediately following packaging but did not result in persistent differences during refrigerated storage. Lower initial a* values of the heart-containing treatments corresponded with differences in calculated myoglobin forms on day 0, when formulations containing beef heart exhibited greater metmyoglobin and lower deoxymyoglobin percentages than control. A greater proportion of oxidized metmyoglobin in the heart-containing formulations may have contributed to the lower initial a* values. As storage progressed, treatment differences in a* were no longer detected, indicating that the initial effect of beef heart incorporation on a* did not persist under vacuum-packaged storage conditions. Similar results have been reported in vacuum-packaged ground beef with oxygen-barrier properties influencing the extent of surface color change [8,21].

**Figure 4:**
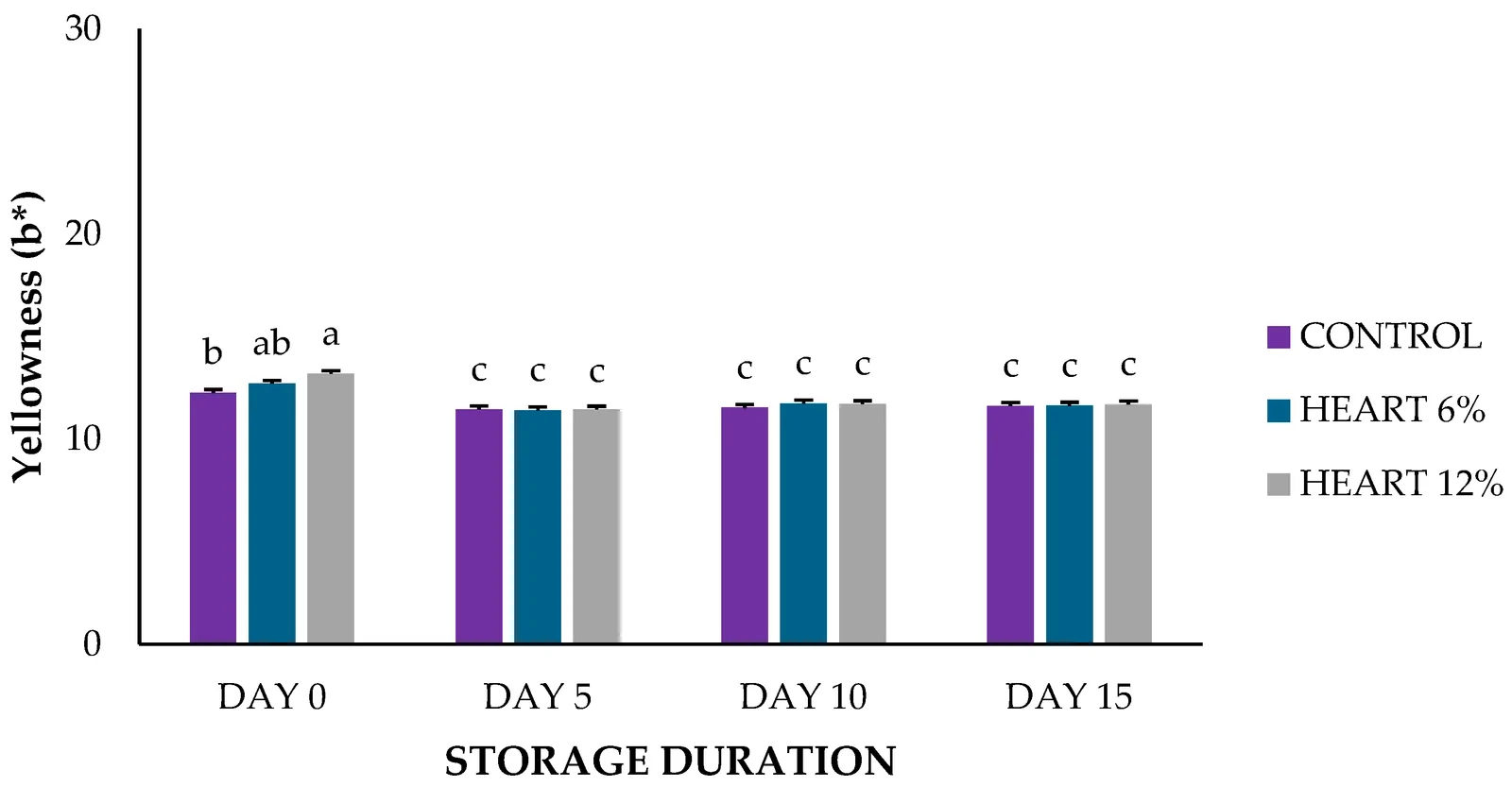
Interaction of ground beef formulation treatment × storage duration on yellowness (b*) values. ^a–c^ Bars without common superscripts differ (*p* < 0.05).

Yellowness (b*) was also affected by treatment × storage day interaction (*p* < 0.05; Figure 4). On day 0, heart 12% had the greatest b* value compared to the control which exhibited the lowest b* value (*p* < 0.05). Following five days of storage, b* values decreased in all treatments and no differences were detected among formulations (*p* > 0.05). Greater initial b* values observed in heart-containing formulations correspond with their lower a* values, indicating subtle differences in surface color immediately after packaging. A decline in b* and absence of treatment differences after day 0 suggest that storage under vacuum altered the color of all formulations similarly once low-oxygen conditions became established. Previous studies have also reported storage-related changes in b* values of vacuum-packaged ground beef, demonstrating that the effect of formulation or packaging may be most apparent early in storage before the package environment has fully equilibrated [21].

**Figure 5:**
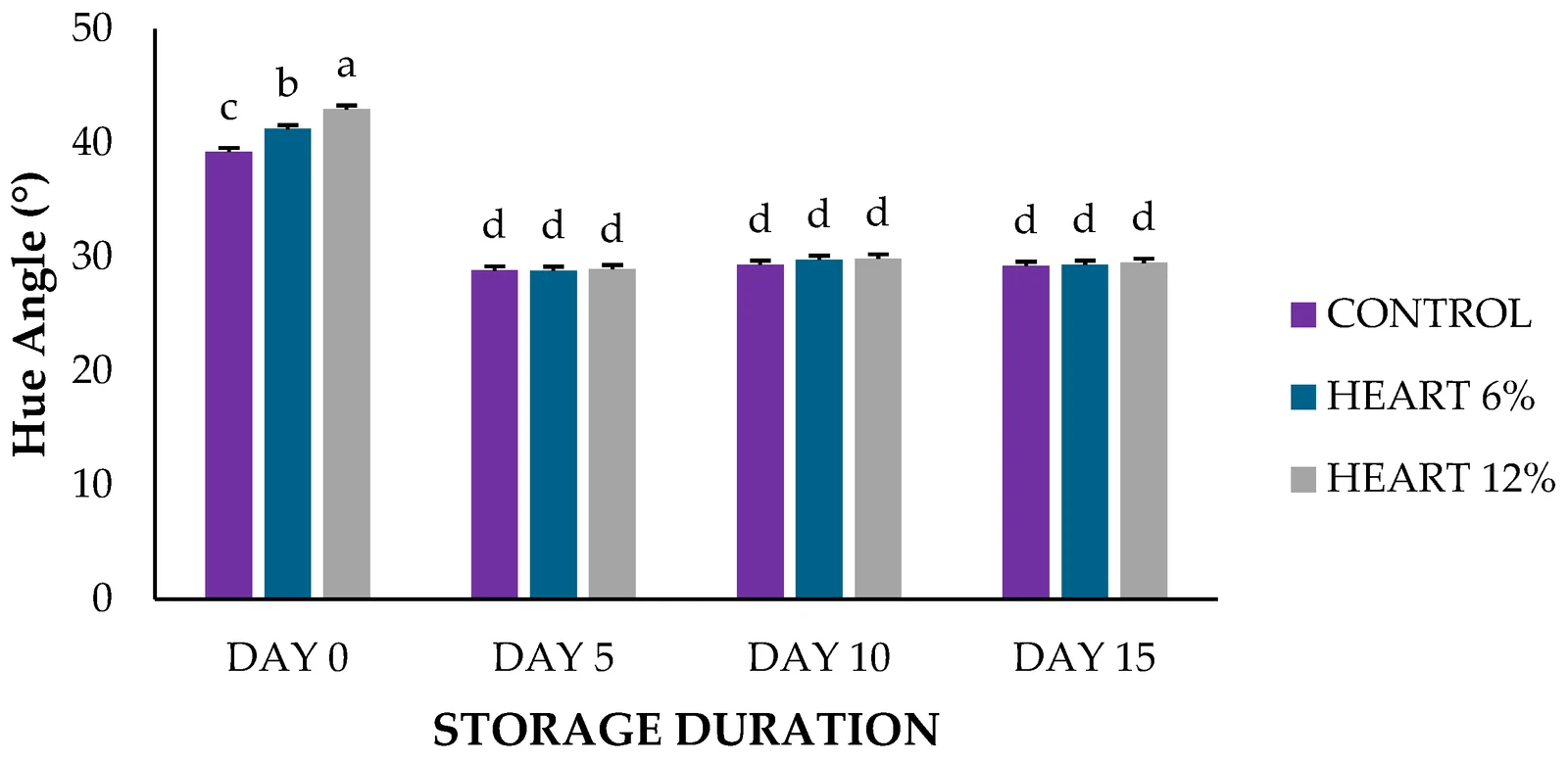
Interaction of formulation treatment × storage duration on hue angle of ground beef. ^a–d^ Bars without common superscripts differ (*p* < 0.05).

Hue angle was affected by a treatment × storage day interaction (*p* < 0.05; Figure 5). On day 0, hue angle increases with addition of heart, with heart 12% exhibiting the greatest value, followed by heart 6% while control had the lowest hue angle value (*p* < 0.05). Hue angle decreased in all treatments by day 5 and remained similar amongst treatments through the duration of storage (*p* > 0.05). Since greater hue angles indicate movement away from the red axis toward a more yellow or brown appearance, these results are consistent with the lower a* and greater b* values observed in formulations containing heart [11]. Similarly, changes in instrumental color have previously been reported with increasing beef heart in ground beef patties, suggesting that incorporation of cardiac muscle can alter the amount of myoglobin present resulting in varying surface color [10]. However, under vacuum-packaged conditions the influence of formulation on hue angle was temporary.

**Figure 6:**
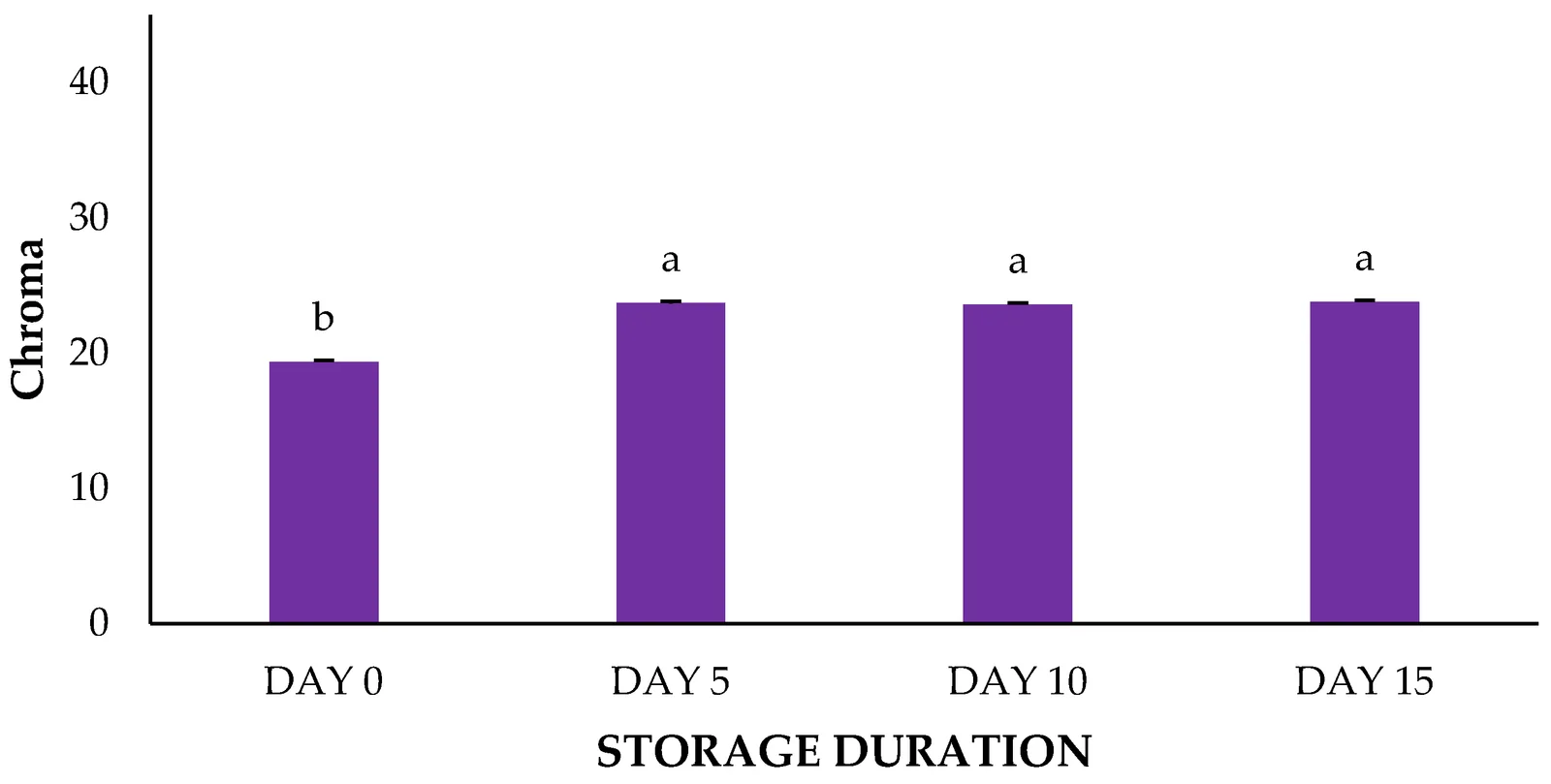
Storage duration effect on chroma values of ground beef. ^a–b^ Bars without common superscripts differ (*p* < 0.05).

Storage day affected chroma (*p* < 0.05; Figure 6), whereas treatment (*p* > 0.05) and the treatment × storage day interaction were not significant (*p* > 0.05). Color saturation increased from day 0 to day 5 and remained relatively stable throughout the remainder of storage (*p* > 0.05). With no treatment effects presented this indicates that beef heart addition did not alter the intensity of surface color under vacuum packaging, despite differences in a* and hue angle immediately following packaging. Instead, the increase in chroma during storage likely reflects the reduced pigment state characteristic of vacuum-packaged beef as residual oxygen was depleted [5,14].

**Figure 7:**
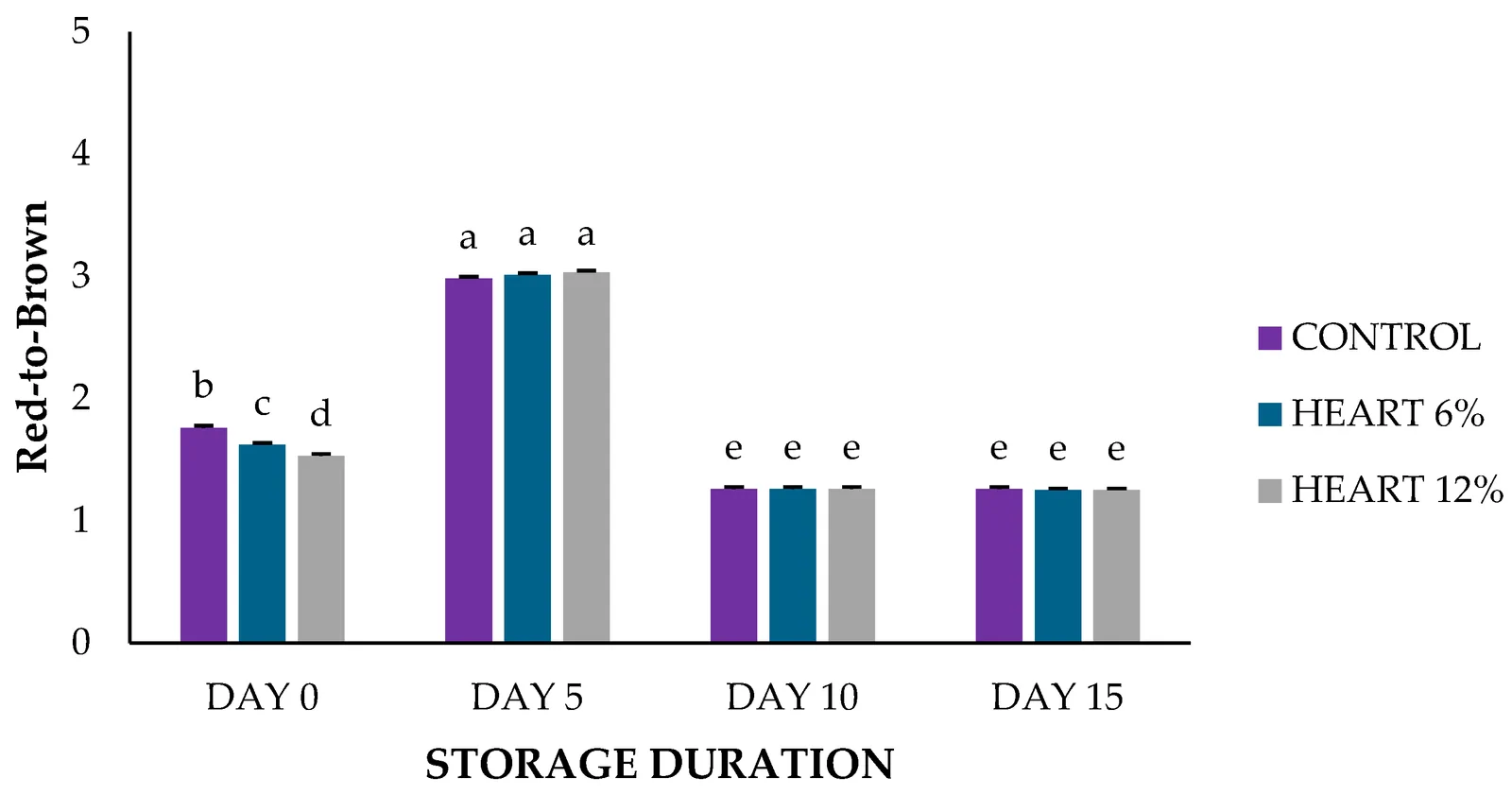
Interaction of formulation treatment × storage duration on red-to-brown ratio of ground beef. ^a–e^ Bars without common superscripts differ (*p* < 0.05).

Additionally, a treatment × storage day interaction affected the red-to-brown (RTB) ratio (*p* < 0.05; Figure 7). On day 0, the control exhibited the greatest RTB ratio, followed by heart 6% whereas heart 12% (*p* < 0.05) had the lowest values. Values increased in all treatments by day 5 (*p* > 0.05) with no differences among formulations. RTB values declined by day 10 and remained similar through day 15 (*p* > 0.05). RTB ratio is widely used as an indicator of surface discoloration as it reflects changes in light reflectance associated with myoglobin chemistry [5]. A lower ratio observed with increasing heart is consistent with the instrumental color measurements demonstrating that ground beef formulated with beef heart primarily influenced the initial appearance of ground beef immediately after packaging.

### 3.3. Calculated Relative Myoglobin Forms

Since meat color is determined by the relative proportions of myoglobin pigments, evaluation of calculated myoglobin forms provides additional insight into the changes observed in instrumental color. Oxymyoglobin imparts the bright cherry-red color associated with fresh beef, deoxymyoglobin produces the purple-red appearance characteristic of vacuum-packaged meat, and metmyoglobin is associated with brown discoloration resulting from pigment oxidation [5,22]. Since pigment distribution is strongly influenced by oxygen availability, vacuum packaging promotes formation of deoxymyoglobin while limiting oxidative discoloration [15].

**Figure 8:**
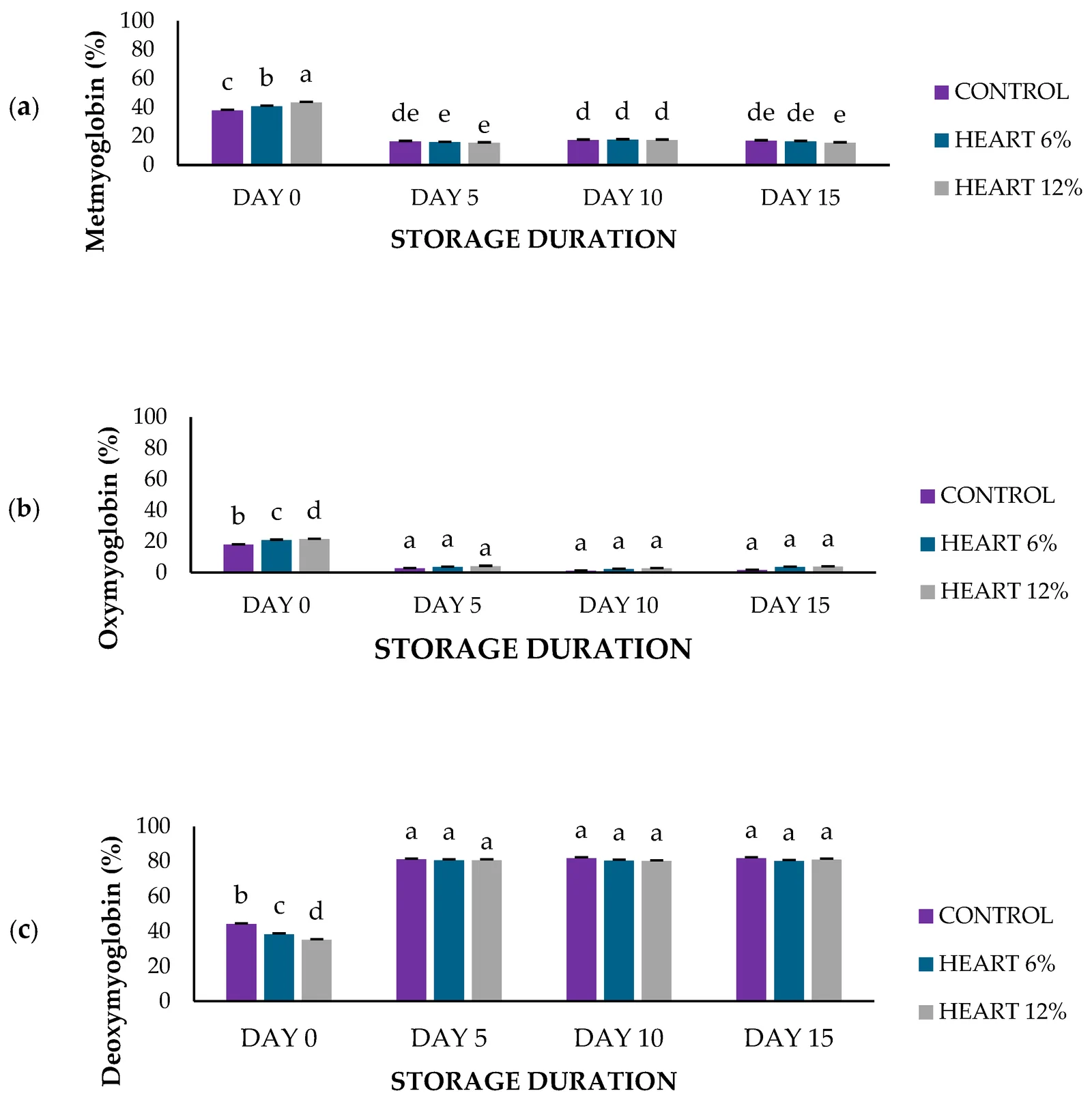
(**a**) Interaction of ground beef formulation treatment × storage duration on relative metmyoglobin values. (**b**) Interaction of ground beef formulation treatment × storage duration on relative metmyoglobin values. (**c**) Interaction of ground beef formulation treatment × storage duration on relative deoxymyoglobin values. ^a–e^ Bars without common superscripts differ (*p* < 0.05).

Treatment × storage day interactions were observed for metmyoglobin (*p* < 0.05; Figure 8a), deoxymyoglobin (*p* < 0.05; Figure 8b), and oxymyoglobin (*p* < 0.05; Figure 8c). On day 0, the 12% heart treatment exhibited the greatest metmyoglobin, followed by heart 6% and the control (*p* < 0.05). Similarly, control exhibited the greatest deoxymyoglobin percentage followed by heart 6% and then heart 12% (*p* < 0.05). For oxymyoglobin, both treatments containing heart exhibited greater percentages than the control (*p* < 0.05) while heart 6% and heart 12% did not differ from each other (*p* > 0.05). By day 5, metmyoglobin and oxymyoglobin decreased with deoxymyoglobin increasing in all treatments (*p* < 0.05). No treatment differences were observed for any myoglobin form from day 5 through 15 (*p* > 0.05). These results indicate that although beef heart influenced the initial distribution of myoglobin pigments, pigment composition became similar among formulations once vacuum storage was established.

Changes observed in calculated myoglobin forms closely align with the instrumental color results. Formulations containing beef heart exhibited lower a* values, greater hue angle, and lower red-to-brown ratios immediately following packaging, corresponding with greater metmyoglobin and lower deoxymyoglobin percentages on day 0. However, these differences largely disappeared after five days of refrigerated storage as pigment distributions became similar among treatments. This indicates that the initial influence of beef heart inclusion on surface appearance was temporary under vacuum-packaged conditions.

Collectively, the instrumental color and calculated myoglobin results demonstrate that vacuum packaging exerted a greater influence on pigment chemistry than formulation during refrigerated storage. Although beef heart altered the initial distribution of myoglobin pigments immediately following packaging, the low-oxygen environment promoted deoxymyoglobin formation across all treatments, minimizing differences in surface color after day 5. These findings indicate that the initial color differences associated with beef heart did not persist throughout refrigerated storage under vacuum packaging.

### 3.4. Thiobarbituric Acid-Reactive Substances

**Figure 9:**
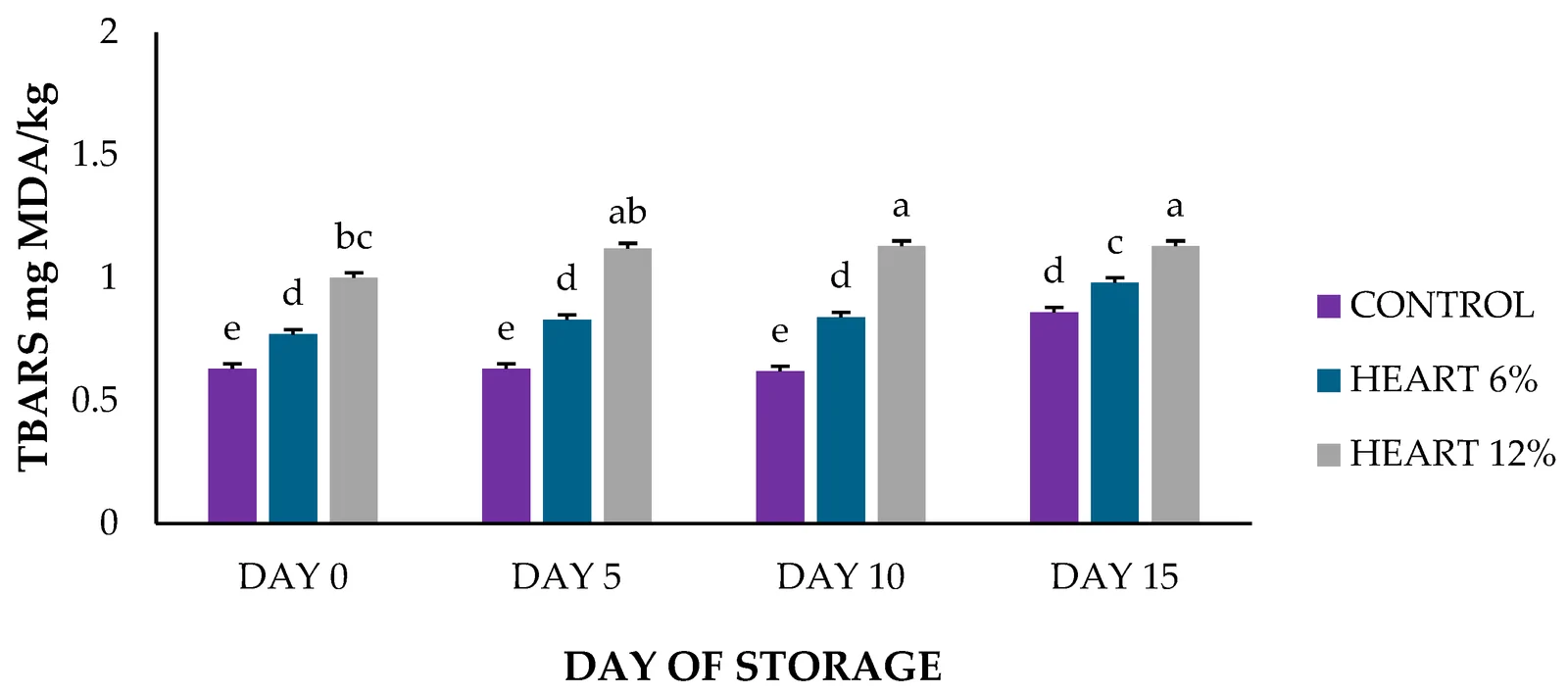
Interaction of ground beef formulation treatment × day of storage on lipid oxidation. ^a–e^ Bars without common superscripts differ (*p* < 0.05).

Lipid oxidation is one of the primary factors limiting the shelf life of fresh meat because it contributes to the development of rancid flavors, off-odors, discoloration, and reduced consumer acceptability [18,23]. TBARS was measured through the quantification of malonaldehyde (MDA) within ground beef samples throughout the 15-day storage period (Figure 9). A treatment × storage day interaction was observed (*p* < 0.05). Across all storage days, heart 12% exhibited the greatest amount of oxidation followed by heart 6% and the control. It is well documented that TBARS values increase over storage time which is consistent with the results of the current study [21]. Values increased throughout storage in all treatments indicating progressive lipid oxidation over time (*p* < 0.05).

Greater TBARS values were observed with increasing heart formulation use are consistent with the compositional differences between beef heart and skeletal muscle. On day 0, TBARS values were greater in samples containing 12% beef heart when compared with ground beef containing 6% beef heart and the control (*p* < 0.05). Additionally, although lower than 12% treatment, samples containing 6% beef heart had greater values on day 0 than the control. The increase on day 0 is likely because the beef heart is composed of highly oxidative cardiac muscle that is rich in myoglobin and heme-containing proteins [2]. It is plausible the increase in TBARS values on day 0 reflects the greater abundance of heme prooxidants that can promote lipid oxidation [18,24,25].

Although TBARS values increased as heart percentage increased, it is important to note that although heart 12% was more oxidized than other treatments, measured values do not exceed the 2 mg/kg threshold [26]. While this value should not be interpreted as an absolute sensory threshold due to the difference in experimental conditions, the relatively low TBARS concentrations observed in the current study suggest that lipid oxidation remained below levels previously associated with substantial oxidative flavor deterioration in beef [26]. This suggests that greater oxidation measured in formulations containing beef heart was unlikely to result in off-flavors during the 15-day storage period. Similar findings have demonstrated that vacuum packaging slows oxidative deterioration by limiting oxygen availability during storage [15,21]. While vacuum packaging did not eliminate formulation-related differences in oxidative stability, it likely slowed the amount of lipid oxidation during storage. Current findings indicate that beef heart increased lipid oxidation during refrigerated storage, particularly at the 12% level. However, under vacuum-packaged conditions, the amount of oxidation remained limited and did not reach concentrations commonly associated with deterioration. Therefore, although beef heart increased oxidative susceptibility, the practical implications for refrigerated shelf life under vacuum packaging appear to be minimal.

### 3.5. Study Limitations

The current study has a few limitations that should be considered when interpreting these findings. Evaluation was limited to physicochemical indicators of storage stability during 15 days of refrigerated vacuum storage, and microbiological analyses and sensory evaluations were not conducted. Therefore, the results should not be interpreted as establishing microbiological product safety or sensory acceptability throughout refrigerated storage. Additionally, although differences in muscle composition and postmortem biochemical processes may contribute to the responses observed in pH and oxidative stability, these mechanisms were not directly evaluated. Future research incorporating microbiological analyses, sensory evaluation, and additional biochemical measurements would provide a more comprehensive evaluation of the storage stability of ground beef formulations containing beef heart.

## 4. Conclusions

Incorporation of up to 12% beef heart influenced the initial physicochemical characteristics of vacuum-packaged ground beef but had limited influence on product stability throughout refrigerated storage. Formulations containing beef heart exhibited lower initial a*, greater hue angle, and altered distributions of myoglobin forms immediately following packaging, indicating that incorporation of cardiac muscle influenced initial pigment characteristics. However, these differences largely disappeared after five days of refrigerated storage as the vacuum environment promoted a stable deoxymyoglobin state across all formulations. Although oxidation increased with beef heart, particularly at the 12% inclusion level, values remained below concentrations commonly associated with the development of perceptible oxidative rancidity during the 15-day storage period. Likewise, treatment differences in pH were small and unlikely to have substantially influenced product stability.

Collectively, these findings demonstrate that the effects of vacuum packaging on pigment stability during refrigerated storage outweighed the influence of beef heart incorporation. While beef heart increased oxidative susceptibility, the low-oxygen packaging environment effectively minimized long-term differences in instrumental color and myoglobin chemistry, resulting in similar color stability among treatments after the initial storage period. These results indicate that incorporating up to 12% beef heart into ground beef is compatible with commercial vacuum-packaging systems and supports the development of nutrient-dense, value-added beef products that improve utilization of edible beef co-products without substantially compromising refrigerated shelf-life characteristics. Overall, these findings demonstrate the potential for beef heart to be incorporated into ground beef formulations while maintaining physicochemical quality during refrigerated vacuum storage, particularly at the 6% inclusion level. Incorporation of beef heart into ground beef may provide an opportunity to develop value-added products that utilize a greater proportion of edible components from the beef carcass.

## Data Availability

The original contributions presented in this study are included in this article; further inquiries can be directed to the corresponding author.

## Abbreviations

The following abbreviations are used in this manuscript:

AMSA	American Meat Science Association
DMb	Deoxymyoglobin
EDTA	Ethylenediaminetetraacetic acid
MDA	Malondialdehyde
MMb	Metmyoglobin
OMb	Oxymyoglobin
RTB	Red-to-brown ratio
TBARS	Thiobarbituric acid reactive substance
USDA	United States Department of Agriculture
